# Measuring and reducing biofilm in mosquito rearing containers

**DOI:** 10.1186/s13071-020-04315-8

**Published:** 2020-09-02

**Authors:** Catherine M. Hunt, C. Matilda Collins, Mark Q. Benedict

**Affiliations:** 1 Centers for Disease Control and Prevention (CDC), 1600 Clifton Road, NE, Atlanta, GA 30329-4027 USA; 2 Centre for Environmental Policy, Imperial College London, 16-18 Princes Gardens, London, SW7 1NE UK

**Keywords:** Mosquito rearing, Biofilm, Bacteria, Aquaculture, Pathogens

## Abstract

**Background:**

Mosquito rearing containers contain organic-rich water that nourishes numerous bacteria, some of which are capable of forming biofilms. Biofilm is broadly an extracellular polymeric matrix (EPS) in which living bacteria occur, and the accumulation of biofilm is possible during routine stock-keeping as most of these containers are re-used. Whether biofilm has an effect on the mosquito rearing is not a question that has been investigated, nor have measures to reduce biofilm in this context been systematically studied.

**Methods:**

We measured biofilm accumulation in standard rearing containers by staining with crystal violet and determining the OD using a spectrophotometer. We also treated rearing containers with 0.1% sodium hypochlorite to determine its effectiveness in reducing biofilm abundance. Lastly, we performed an analysis of the relationship between the occurrence of biofilm and the likelihood of microbial blooms that were associated with larval death during trials of larval diets.

**Results:**

We observed that soaking rearing containers overnight in 0.1% sodium hypochlorite greatly reduced biofilm, but we observed no relationship between the use of containers that had not been treated with bleach and subsequent microbial blooms.

**Conclusions:**

Larva rearing leaves detectable biofilm. While we were unable to correlate microbial blooms with the presence of biofilm, as a precaution, we recommend that plastic containers that are re-used be treated with 0.1% sodium hypochlorite occasionally. 
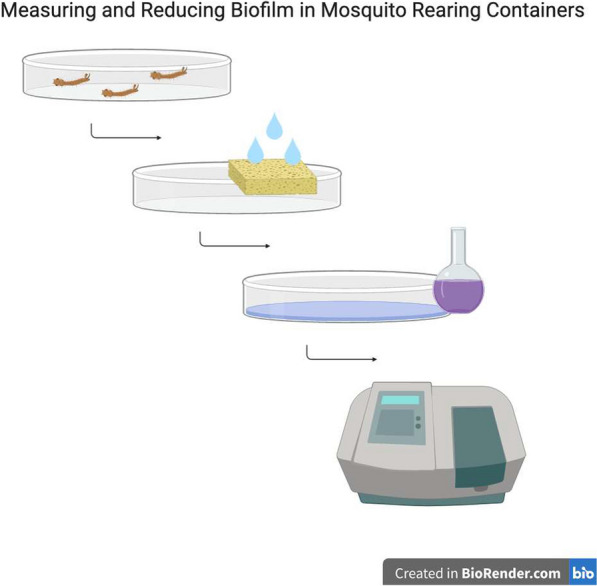

## Background

By far the most common containers used for rearing mosquito larvae are plastic or metal trays. These are usually re-used and may be cleaned in a number of ways including with detergent or water alone, and they are sometimes treated with bleach or autoclaved after washing. In our experience, many insectary staff are reluctant to use detergents for fear of contaminating the containers with any residue that might harm larvae, but we are aware of no systematic efforts to determine how likely this is. Other effects of any change in larval rearing methods are often of concern. For example, two reports have noted that larval exposure to detergents and hydrogen peroxide affected the level of insecticide resistance measured in resulting adults though intergenerational selection for this was not demonstrated [1, 2]. No comparison with the level of trace contamination of cleaners used in mosquito rearing was made.

Because mosquito rearing is not aseptic, introduction of pathogens is always a possibility. In fish aquaculture, which has similar characteristics, when even non-pathogenic opportunistic bacteria become excessively abundant, death can occur [3]. In mosquito larval culture substantial bacterial growth is usually evidenced by extremely turbid water, a foul odor and can result in complete larval death in the container. We will refer to this event simply as a ‘microbial bloom’. The usual method to avoid this is to prevent excess amounts of food and to maintain a larval density that keeps the amount of food per larva in the container from becoming excessive; however, this results in a chronic state of food restriction [4].

Biofilm occurs on surfaces that are exposed to biological material in aqueous liquids. They consist of bacteria embedded in a matrix, the extracellular polymeric substances (EPS), that many bacteria secrete consisting of polysaccharides, lipids, nucleic acids and proteins [5, 6]. The EPS itself is a complex microhabitat that protects and promotes the persistence of the bacteria that reside in it and contribute to its structure. It is difficult to remove, being resistant to desiccation, ultraviolet light and detergents [7].

While biofilms occur in diverse environments such as industrial equipment, human health products and soil, in the context of mosquito rearing, aquaculture provides an analogous source of experience in managing issues associated with biofilm formation. Indeed, previous studies have reported the potential for harm that biofilms present in aquaculture systems. King et al. [3] found fish and human pathogens present in biofilms in aquaculture facilities. Although most of the fish pathogens identified were opportunistic and non-detrimental, three were known to cause significant disease in marine fish populations. Furthermore, Skjermo et al. [8] described the negative effect of microbial growth on the early stages of marine larvae. High larval densities, decay from dead larvae, and low water exchange contributed to intense biofilm formation that ultimately led to increased mortality or inferior adults. Such conditions are reminiscent of typical mosquito larva rearing conditions.

Biofilms can be extremely difficult to remove and physical measures alone, i.e. scrubbing or high-pressure spraying, are not completely effective, often leaving largely invisible source colonies of contaminating bacteria. The use of chemicals, such as sodium hypochlorite, has been shown to significantly reduce the presence of biofilm depending on the substrate [7]. We therefore chose this widely available and inexpensive chemical to determine its effect on biofilm presence and amount in mosquito rearing containers.

We report the results of a simple method to detect biofilm in mosquito rearing containers and the effect of bleaching on removing biofilm. The preliminary results of the relationship between rearing larvae in untreated used larval containers compared to new containers is also reported. The likelihood of microbial blooms, a correlate of biofilm that might cause substantial or total mortality of rearing stock, was investigated. We also analyzed the effect of larval food type, form and concentration and whether the development of biofilm varies between the mosquito species *Aedes aegypti* and *Anopheles gambia*e. We will generally refer to the residual substances on the submerged surfaces of rearing trays and dishes as ‘biofilm.’

## Methods

### Effects of mosquito species, food type and quantity

The rearing methods and experimental material that were analyzed in the observations reported here have been published previously [4]. Briefly, 80 L1 *An. gambiae* ‘G3’ or *Ae. aegypti* ‘New Orleans’ strain larvae were reared in 150 mm Petri dishes containing approximately 100 ml of standard rearing water consisting of 0.3 g of pond salts (API, McLean, VA USA) per liter of type II purified water. After pupation, the containers that were to be reused were scrubbed with a wet sponge, rinsed in type II water and thoroughly air-dried. Alternatively, new containers were used. Four diets were used; two of these were custom formulations of a diet specifically designed for mosquitoes [9]. This diet consists of a 2:2:1 ratio (by weight) of bovine liver powder, tuna meal and Vanderzant vitamin mix. One formulation was prepared at CDC in Atlanta, GA using ‘Now’ brand liver powder (Bloomingdale, IL USA), tuna meal (AA Baits, Rock Ferry, Birkenhead, UK) and Vanderzant vitamin mix (Bio-Serv, Flemington, NJ, USA). The other formulation of the Damiens diet was prepared by Frontier Scientific Services (Newark, DE USA) using defatted, desiccated liver powder (product no. 1320; Frontier Scientific Services), Vanderzant vitamin mix (product no. F8045, Frontier Scientific Services) and the same lot of tuna meal as was used at CDC. The other two diets were commercially available fish foods; TetraMin Plus Flakes (Tetra GmbH, Melle, Germany) and Doctors Foster and Smith Koi Staple diet (Rhinelander, WI USA). Koi pellets were ground in a Miracle Model MR-300 Electric Grain and Flour Mill (Danbury, CT USA) followed by sieving and saving the particles that passed a 600 µm standard sieve. The TetraMin was ground in a Black and Decker ‘SmartGrind’ coffee grinder (Beachwood, OH USA) to a consistency that passed through a 600 µm sieve. These diet types will be referred to as CDC, Frontier, TetraMin, and Koi respectively. Diet was fed at two concentrations, 1.6% or 3.2% on alternate days. We also fed one diet, Koi, as a pellet, the form in which it is supplied, or as a powder. Two pellets (equivalent to 52 mg/dish/day) were fed on alternate days (see [4] for details). There were three dishes for each species, food and concentration.

### Estimating biofilm abundance

A quantitative estimate of the amount of biofilm was made *in situ* by staining with crystal violet (Product no. C0775-25G, Millipore Sigma, Billerica, MA, USA), essentially by the method of O’Toole [10]. A 0.1% solution of crystal violet was prepared, and 10 ml were added to each Petri dish. Dishes were stained with gentle agitation for 15 min and then rinsed 3 times with type II water and air-dried overnight. The bound crystal violet was eluted by adding 20 ml of 30% acetic acid and incubated at room temperature for 15 min. The OD_590_, which is the maximum absorbance of crystal violet, was then measured using a general-purpose UV/VIS spectrophotometer (720 series, Beckman Coulter, Atlanta, GA USA) in 1 cm light path polystyrene cuvettes. We confirmed that maximal absorbance was 590 nm in the 30% acetic acid solution rather than 550 nm as described by O’Toole [10]. Background absorbance was measured on eluant from new dishes and subtracted from the absorbance values of all samples. The spectrophotometer was zeroed against a blank containing 30% acetic acid after approximately every 5 measurements to be certain there was no drift in observations.

### Cleaning method

The effect of overnight bleaching on biofilm abundance was also determined. Thirty previously-used dishes (without knowledge of the specific food type or amount used) were allocated into two equal groups of 15; one group was treated overnight in a solution of 0.1% sodium hypochlorite prepared from an 8.5% sodium hypochlorite solution of concentrated Chlorox (Oakland, CA, USA) bleach diluted in type II water. This formulation of bleach contained no fragrances or sodium hydroxide which are found in some household bleach solutions. After bleach treatment, the dishes were thoroughly rinsed three times in type II water and fully air-dried. The OD_590_ was determined as above.

### Food form

To explore whether the form in which the food was provided might affect the amount of biofilm that formed, we established an orthogonal set of six dishes for each species, half of which were provided with two Koi pellets and half were provided a 1.6% Koi slurry. Dishes were treated with crystal violet as mentioned above, and the OD_590_ was determined. As a side note, we observed that *Ae. aegypti* are active feeders that disintegrated the pellets rapidly whereas the pellets remained largely intact for up to 2 days when placed in dishes containing *An. gambiae* which we thought might affect the amount of biofilm. It should be kept in mind that the weight of Koi food when fed as a pellet was greater than when fed as a slurry.

### Analyses

Statistical analyses were performed using R version 3.5.1 “Feather Spray” [11]. As OD_590_, the measure of biofilm abundance, was a continuous variable, an ANOVA was used. The OD_590_ data were strongly skewed and had a single outlier, these data were ln-transformed for tractability in the analyses. The transformed values of the response variable (optical density) led to substantially improved model fit in all cases. As model fit was good, in no case was there evidence of particular influence by the outlier apparent in the non-transformed data.

Full models were fit with all main effects and interactions and the importance of terms was assessed by stepwise simplification. As the data are low replication, *P* < 0.01 was used as a threshold for assessing the significance of interactions to avoid over-interpretation; *P* < 0.05 was used for main effects. The occurrence (presence/absence) of total larval mortality which indicated microbial blooms as a function of whether the dish was previously used or new was analyzed by Fisher’s exact probability test. The effect of bleaching on the residual biofilm abundance was estimated by adjusted t-test to allow for the substantially different variance observed between the bleached and unbleached groups.

## Results

### Effects of mosquito species, food type and quantity

**Fig. 1:**
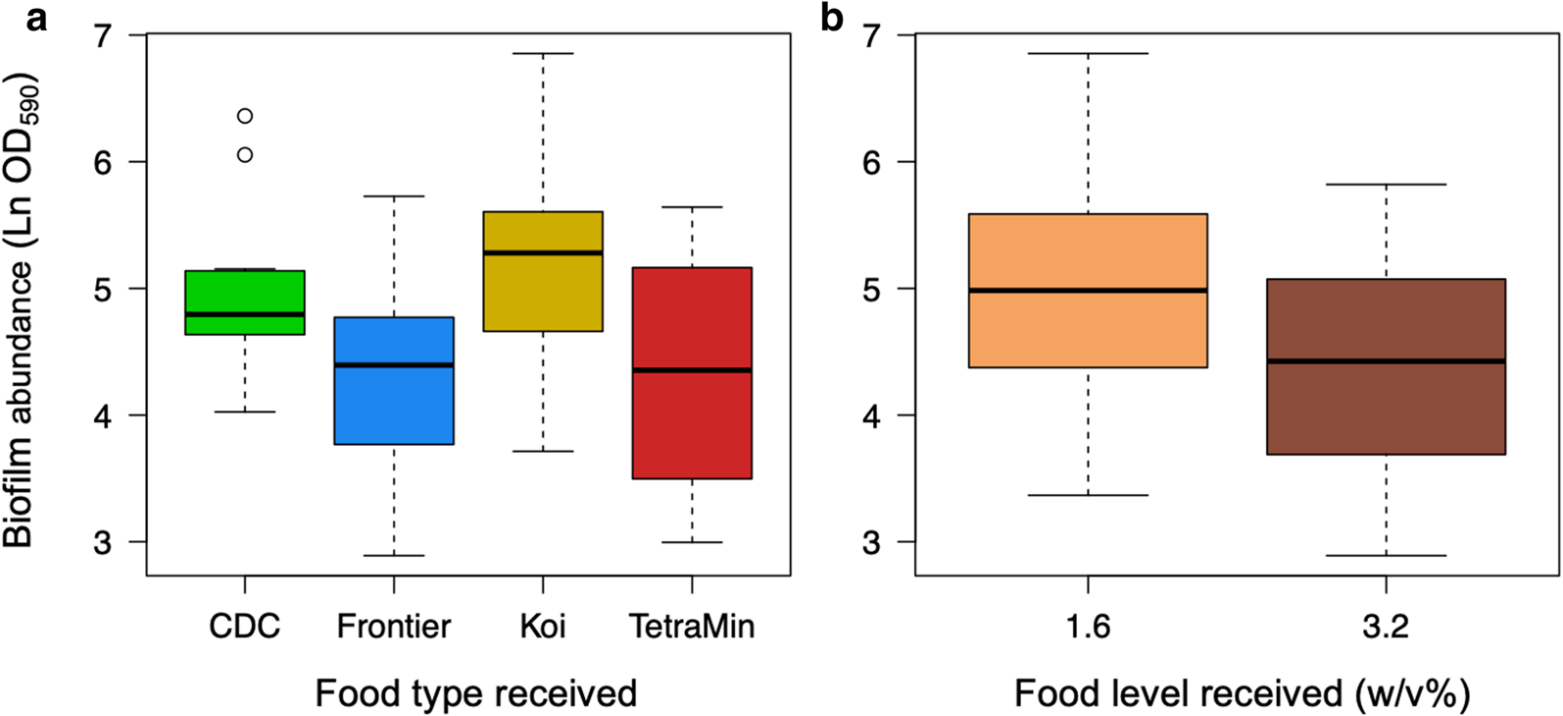
The abundance of biofilm (ln OD_590_) as a function of food type (**a**) and the quantity supplied (% w/v) (**b**)

There was no evidence of interactions between the species of mosquito being reared, the quantity or the type of food found in these data (*P* > 0.01 in all cases) affecting the measured abundance of biofilm. No three-way interaction was detected between the main effects (*F*_(32, 35)_ = 1.75, *P* = 0.18) nor between food type and the dose it was given at (*F*_(35, 38)_ = 0.73, *P* = 0.54) or between food type and the mosquito species (*F*_(38, 41)_ = 1.57, *P* = 0.21). The hint of interaction suggesting that the biofilm abundance was higher at lower dose for *An.* *gambiae* did not pass our threshold for interactions and was potentially affected by the single outlier in the data (*F*_(41, 42)_ = 7.07, *P* = 0.012). Within the main effects, there was also no evidence of variation in biofilm abundance attributable to the species being reared (Mean ± SE) Ln(OD_590_), *Ae. aegypti*: 4.6 ± 0.14, *An. gambiae*: 4.77 ± 0.02, *F*_(42, 43)_ = 0.59, *P* = 0.48). There was, however, significant variation detected as a function of food type with Frontier and Tetramin leading to slightly lower biofilm abundance than the CDC & Koi formulations (*F*_(43, 46)_ = 3.84, *P* = 0.016, Fig. 1). The level of food received was also found to affect the biofilm (*F*_(43, 44)_ = 7.81, *P* = 0.008), though this was in a counter-intuitive way with a higher biofilm being observed at the lower food level (1.6% w/v = 4.9 ± 0.17; 3.2% w/v = 4.38 ± 0.16, Fig. 1).

In all, we observed 12 occurrences of microbial bloom as indicated by foul odor and extreme turbidity that preceded complete larval mortality. The likelihood of this happening was, however, independent of whether dishes had been used previously or were new (Fisher’s exact test, *P* = 0.68).

### Estimating biofilm abundance and cleaning method

**Fig. 2:**
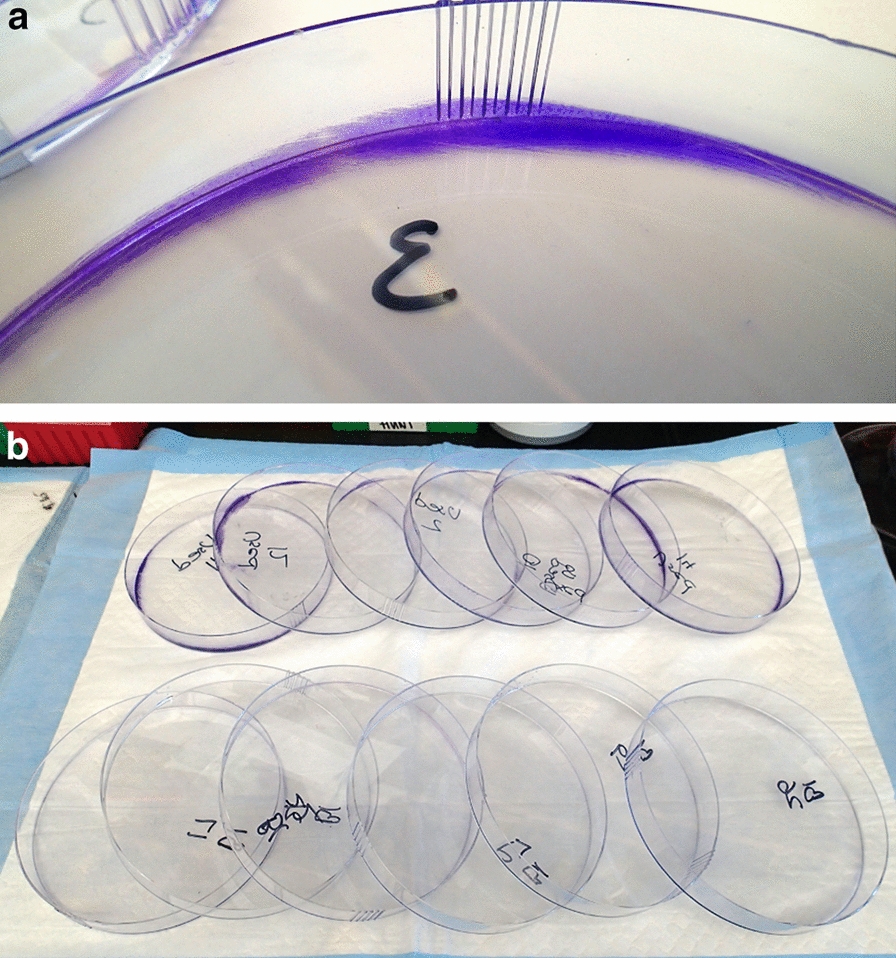
(a) Biofilm detected on ‘cleaned’ plates visualized by staining with crystal violet. (**b**) Previously used dishes that were washed and bleached (top row) versus washed only (bottom row)

Biofilm was readily seen in used dishes after staining with crystal violet (Fig. 2). The residual biofilm abundance on plates was also observed in solution after elution. Bleaching greatly reduced residual biofilm (*t* = 14.06, *df* = 23.05, *P* < 0.001): the mean, untransformed OD_590_ of the bleached group was 38.07 ± 3.82 compared to 397.40 ± 49.54 for the unbleached group.

### Food form

**Fig. 3:**
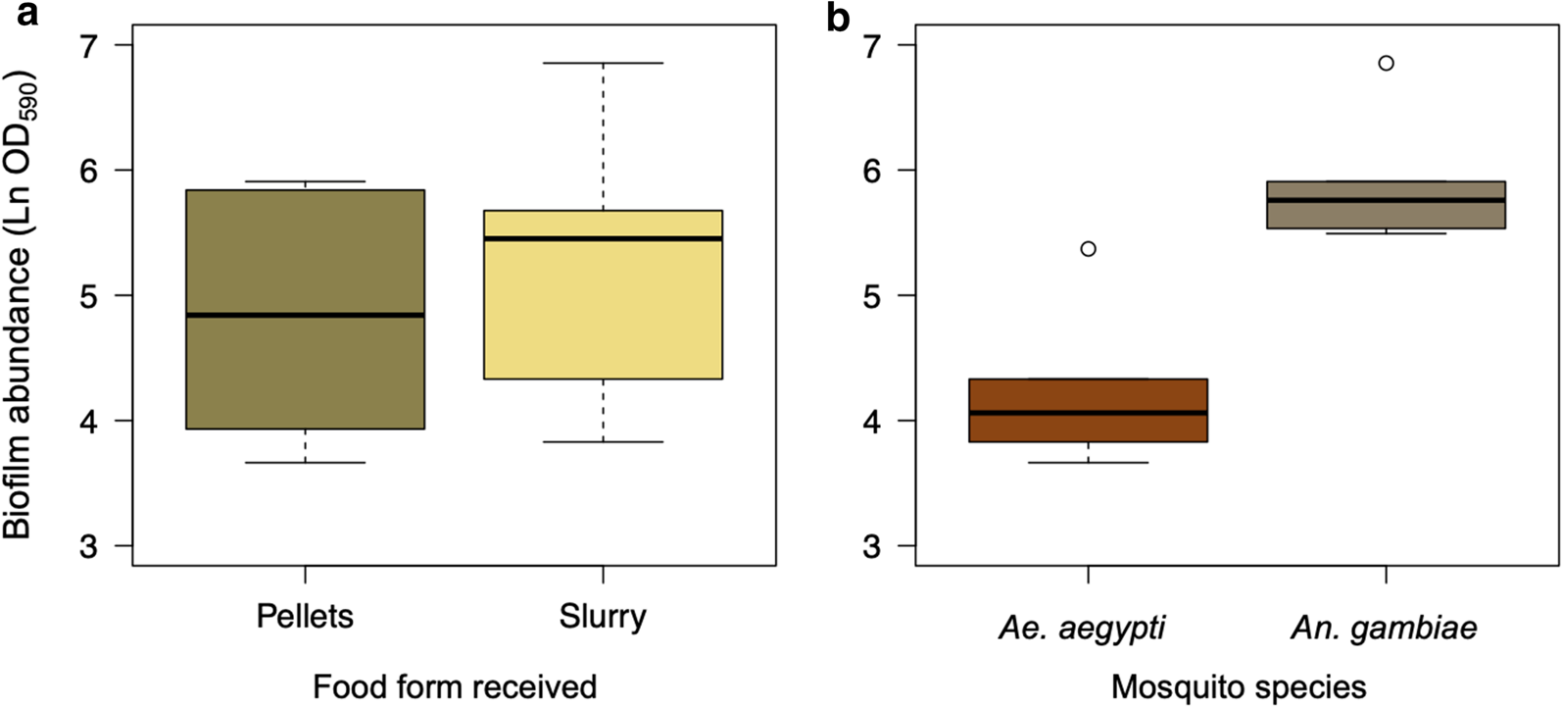
The abundance of biofilm (Ln OD_590_) as a function of the form in which the food was provided (**a**) and the mosquito species being reared (**b**)

In these experiments, two factors were analyzed to determine their effect on the amount of biofilm in the dishes: the species of mosquito and whether the food was fed as a pellet or slurry; any interaction between the species and form of the food was also evaluated. There was no interaction identified and the effect of food form on biofilm amount did not vary with the species being reared (*F*_(8, 9)_ = 0.22, *P* = 0.65, Fig. 3). Nor did the form, whether slurry or a pellet, affect this (*F*_(9, 10)_ = 1.90, *P* = 0.20). The biofilm did vary between species, however, and was substantially greater in the *An. gambiae* than in the *Ae. aegypti* dishes (*F*_(10, 11)_ = 26.44, *P* < 0.001).

## Discussion

Biofilm is a potential source of mosquito rearing difficulties, and even though autoclaving may kill the bacteria within the EPS, the biofilm matrix can persist, promoting subsequent biofilm formation. While we did not observe whether bleaching killed the bacteria within the biofilm, it greatly reduced the amount of detectable biofilm based on the crystal violet assay and it is reasonable to suppose that it also reduced the viability and persistence of associated bacteria. Among several chemical treatments tested, bleach has been observed to be one of the most effective methods to remove biofilm and is widely used for this purpose [7]. While we did not observe an association between microbial blooms and whether dishes had been used previously or not, which we interpret as correlating with more biofilm, it seems to be a reasonable precaution to treat rearing containers with bleach occasionally. It is possible that a larger number of replicates over time might have revealed an effect of some factors that were insignificant here and is something could be explored in the future.

The data presented here should be interpreted cautiously. Even though the cleaning was considered well done, biofilm was still detectable in the crevices. Therefore, there is a relationship between the efforts of the individual cleaning the dishes and the amount that will persist, and even though biofilm was most evident in crevices, we also observed crystal violet staining generally across the flat surfaces of used dishes even where they appeared clean. The ability of the person cleaning the container to see obvious film may be the reason biofilm was lower in dishes that had contained *An. gambiae* fed at the 3.2% rate than in the 1.6%. In spite of more food being provided, and 3.2% is quite high, there was less residual biofilm. We speculate that because biofilm was easier to see at the higher food rate, it was also easier to detect and thoroughly remove. Because of the variability observed, it would be beneficial to have a standard operating procedure in place to minimize inconsistencies in pan washing or to make bleach treatment a standard procedure.

Apart from the cleanliness of rearing containers, it is important to consider other factors attributing to microbial blooms. Water quality (particularly oxygen content), temperature changes, and food sources are common causes of microbial blooms in aquaculture systems [12]. Further, the type and amount of food used in mosquito culture contributes to water quality; therefore, it is critical to select a diet that is compatible with overall mosquito health and survival [4].

The difference we observed in biofilm abundance between the species being reared may have its source in the feeding habits of these particular species. The voracious nature of *Ae. aegypti* could affect this in two ways: first, it may be that they consume the food so rapidly that biofilm formation is inhibited by lack of available nutrients, or secondly, they may also consume the biofilm itself by scraping and thus reduce it. These two points are not mutually exclusive, and both may act at the same time. The more delicate *An. gambiae* is known to have higher mortality under similar rearing conditions [4] and the nutrients made available through decomposition of larvae may themselves promote biofilm formation.

## Conclusions

These simple observations provide insight into factors that affect biofilm formation in mosquito cultures. Simple bleach treatment seems to be a reasonable precaution to minimize it when using containers that will withstand this rather harsh oxidizer.

## Data Availability

The datasets generated during and/or analyzed during the present study are available from the corresponding author upon reasonable request.

## Abbreviations

ANOVA: analysis of variance
EPS: extracellular polymeric substances
L1: first-stage larvae
OD: optical density
